# Brain-derived neurotrophic factor associated with kidney function

**DOI:** 10.1186/s13098-023-00991-5

**Published:** 2023-02-13

**Authors:** Cheng-Yueh Hsu, Wayne Huey-Herng Sheu, I-Te Lee

**Affiliations:** 1 Medical Education Department, Linkou Chang Gung Memorial Hospital, Taoyuan City, 33305 Taiwan; 2 Department of Internal Medicine, Taipei Veterans General Hospital, Taipei, 11221 Taiwan; 3 School of Medicine, National Yang Ming Chiao Tung University, Taipei, 11221 Taiwan; 4 Division of Endocrinology and Metabolism, Department of Internal Medicine, Taichung Veterans General Hospital, No. 1650 Taiwan Boulevard, Sect. 4, Taichung, 40705 Taiwan; 5 School of Medicine, Chung Shan Medical University, Taichung, 40201 Taiwan

**Keywords:** Brain-derived neurotrophic factor, Chronic kidney disease, Fasting, Oral glucose tolerance test

## Abstract

**Background:**

We examined the relationship between brain-derived neurotrophic factor (BDNF) and chronic kidney disease (CKD).

**Methods:**

First, a cross-sectional study was conducted in 480 participants without known diabetes. An oral glucose tolerance test (OGTT) was administered after overnight fasting, and blood samples were collected at 0, 30, and 120 min. Second, a total of 3003 participants were enrolled for the case–control genetic analysis. After assigning them to a case or a control group based on age and CKD status, we investigated the association between BDNF gene variants and susceptibility to CKD.

**Results:**

A higher fasting serum BDNF quartile was significantly associated with a lower prevalence of CKD (P value for trend  < 0.001). Based on the receiver operating characteristic analysis, the fasting BDNF level had a larger area under the curve for differentiating CKD (0.645, 95% CI 0.583‒0.707) than the BDNF levels at both 30 min (0.547, 95% CI 0.481‒0.612) and 120 min (0.598, 95% CI 0.536‒0.661). A significantly lower CKD prevalence (odds ratio = 0.30, 95% CI 0.12‒0.71) was observed in the highest quartile of fasting BDNF level than that in the lowest quartile, whereas no interquartile differences were observed for BDNF levels determined at 30 or 120 min during the OGTT. Furthermore, BDNF-associated variants, including rs12098908, rs12577517, and rs72891405, were significantly associated with CKD.

**Conclusions:**

The BDNF level at fasting, but not at 30 and 120 min after glucose intake, was an independent indicator of CKD. In addition, significant associations were observed between three BDNF gene variants and CKD.

**Supplementary Information:**

The online version contains supplementary material available at 10.1186/s13098-023-00991-5.

## Introduction

Chronic kidney disease (CKD) is associated with a high risk of mortality and disability [1, 2]. CKD has become a global health burden, and the assessment of its risk factors is important to prevent CKD-associated complications [2–5]. Dysregulation of energy homeostasis and insulin resistance play an important role in the development of CKD and are associated with further morbidity in CKD patients [6, 7]. Hyperglycemia leads to an extra energy expenditure to meet the demand of increased tubular reabsorption [8]. Accordingly, alterations in mitochondrial activity, as reported in primary human glomerular mesangial cells cultured in hyperglycemic conditions, have been shown to contribute to insulin resistance and CKD pathogenesis [9–11]. In turn, CKD can induce skeletal muscle wasting, which may prompt insulin resistance to further aggravate CKD [8, 12].

Brain-derived neurotrophic factor (BDNF) is a member of the neurotrophin family associated with energy homeostasis [13, 14]. Changes in serum BDNF levels in response to oral glucose intake were reported to be associated with body weight [15]. Huber et al. [16] reported that BDNF might be involved in renal tubulogenesis and that tyrosine kinase receptor B (TrkB), the BDNF receptor, is expressed during human kidney development. In a study involving a rodent model, BDNF prevented glomerular podocyte damage through TrkB signaling [17]. Of note, a low circulating BDNF level was reported to be a significant predictor of incident CKD in a longitudinal study [18]. An inverse correlation was established between CKD-related pro-inflammatory cytokine production and BDNF expression [6, 19]. However, the extent of the association between serum BDNF levels and the prevalence of CKD remains unclear.

A study in young healthy volunteers showed that insulin resistance induced by a intralipid/heparin infusion elicits a decrease in serum BDNF level [20]. BDNF has shown to protect endothelial function, and a decrease in serum BDNF might be associated with chronic inflammation and long-term mortality [19, 21]. The interplay between glucose regulation and BDNF is suggested by evidence that the serum BDNF levels measured during an oral glucose tolerance test (OGTT) is a better predictor of cardiovascular outcomes than the fasting BDNF level [22]. Insulin resistance indices derived from the OGTT are helpful for evaluating insulin sensitivity in patients with CKD but are not significantly associated with long-term mortality [23]. We therefore hypothesized that serum BDNF would be inversely associated with the prevalence of CKD. Interestingly, several single-nucleotide polymorphisms (SNPs) in the BDNF gene were associated with muscle fiber composition and cardiovascular outcomes [24–26]. However, the potential impact of BDNF-associated SNP on CKD remains largely undetermined. Based on the above considerations, the present work includes two distinct analyses. First, we examined the correlation between serum BDNF levels, both at fasting and after oral glucose intake, and CKD prevalence. Second, we examined the potential relationship between BDNF SNPs and CKD.

## Materials and methods

### Part 1: analysis of the correlation between serum BDNF levels and CKD

#### Participants and study design

In this cross-sectional study, 480 adult participants without known diabetes mellitus (DM) were enrolled between January 01, 2011 and January 31, 2015. After anthropometric measurements, a 75-g OGTT was performed for 2 h. This study was approved by the Institutional Review Board of Taichung Veterans General Hospital and complies with the guidelines of the Declaration of Helsinki. All participants provided written informed consent when recruited.

#### Sample collection

Blood samples were collected at 0 (fasting status), 30, and 120 min during the OGTT. Fasting blood samples were used to measure BDNF, glucose, hemoglobin A1c (HbA1c), creatinine, and the lipid profile. Blood samples at 30 and 120 min were used to measure BDNF, glucose, and insulin levels. The area under the curve (AUC) of BDNF, as well as glucose and insulin, was calculated based on the data at 0, 30, and 120 min. A morning urine sample was collected for the measurement of albumin and creatinine to calculate the urine albumin-creatinine ratio (UACR).

#### Biochemical assessments

Free BDNF in serum was measured by an immunoassay kit (R&D Systems, Minneapolis, USA) with an intra-assay coefficient of variation (CV) of 6.2% and an inter-assay CV of 8.1%. Plasma glucose was measured by the glucose oxidase–peroxidase method (Wako Diagnostics, Tokyo, Japan). Insulin was measured using commercial kits (Roche Diagnostics GmbH, Mannheim, Germany). The homeostasis model assessment of insulin resistance (HOMA-IR) index was calculated as fasting insulin (mU/L) × fasting glucose (mmol/L)/22.5 [27]. HbA1c levels were measured using boronate affinity high-performance liquid chromatography (NGSP certified, Primus Corp., Kansas City, MO, USA). C-reactive protein (CRP) was assessed using an immunochemical assay involving purified duck IgY (ΔFc) antibodies (Good Biotech Corp., Taichung, Taiwan). Serum levels of creatinine and lipids were measured by commercial kits (Beckman Coulter, Fullerton, USA). The estimated glomerular filtration rate (eGFR) was calculated as 186 × (serum creatinine [mg/dL])^−1.154^ × (age [years])^−0.203^ (× 0.742, if female) according to the Modification of Diet in Renal Diseases equation [28]. CKD was defined as eGFR < 60 mL/min/1.73 m^2^. Urinary albumin levels were assessed using the polyethylene glycol enhanced immunoturbidimetric method (Advia 1800, Siemens, New York, USA). The UACR was calculated as the ratio of urine albumin (mg) to urine creatinine (g) [28]. Hypertension was defined as systolic blood pressure  ≥ 140 mmHg, diastolic blood pressure  ≥ 90 mmHg, or a history of antihypertensive medication use.

### Part 2: genetic analysis of the association between BDNF SNPs and CKD

#### Subjects and study samples

We enrolled 3003 participants in the genetic study at Taichung Veterans General Hospital. Blood samples were collected for biochemical analyses and DNA preparation. Genomic DNA was extracted from peripheral leukocytes using a QIAamp DNA Blood Mini Kit (Qiagen, Valencia, USA). Genotyping was performed with the Illumina 200 K Metabochip following the manufacturer’s protocol (Illumina, San Diego, USA) [29].

#### BDNF-associated SNPs

We selected five BDNF-associated SNPs ‒rs12098908, rs12577517, rs6265, rs77351929, and rs72891405‒ and examined the association between their genotypes and CKD. Genotyping was performed successfully for 2901 (96.6%) participants at rs12098908, 2995 (99.7%) participants at rs12577517, 2992 (99.6%) participants at rs6265, 2945 (98.1%) participants at rs77351929, and 2997 (99.8%) participants at rs72891405.

#### Definition of case and control groups

The decline in eGFR that occurs with normal aging correlates with a higher prevalence of CKD at an older age. Therefore, a participant might not have CKD at a young age but have CKD at an older age. To effectively investigate the association between BDNF gene variants and susceptibility to CKD, we grouped participants with CKD younger than 60 years of age into the case group and participants without CKD 60 years of age and older into the control group. Afterwards, we conducted logistic regression analyses of all 3003 participants to examine the effect of the risk alleles on CKD in dominant, recessive, and additive models.

### Statistical analysis

Continuous variables are presented as the mean (standard deviation), and categorical variables are presented as numbers (percentage). To assess differences between the participants with CKD and those without CKD, an independent t test was used to examine continuous variables, and the chi-squared test was used to examine categorical variables, including genotypes. The correlation coefficient between eGFR and BDNF was determined using Pearson’s correlation test. Receiver operating characteristic (ROC) analysis was performed to differentiate CKD by BDNF profile levels during the OGTT.

To investigate whether there is a trend in CKD prevalence across different fasting BDNF levels, we divided all the enrolled participants into four quartiles according to their BDNF levels. To evaluate the relationship between fasting BDNF levels and CKD, we used logistic regression models to estimate the odds ratio (OR) of CKD and the 95% confidence interval (CI) of the three higher fasting BDNF quartiles compared to the lowest fasting BDNF quartile after adjusting for different potential confounding variables. The same trend analysis was also used to evaluate differences across quartiles according to the participants’ BDNF levels at 30 min and at 120 min during the OGTT. In addition to the crude model, we included age (a continuous variable) and sex (a categorical variable) in Model 2 of the regression analyses. In Model 3, we included all the risk factors assessed to evaluate differences across BDNF quartiles. However, to avoid overadjustment, hypertension, but not systolic or diastolic blood pressure, was included in the regression analyses. Similarly, we did not include glucose or HDL cholesterol in the regression analyses because HbA1c, total cholesterol, and triglycerides were included.

In the genetic study, regression analyses were used to evaluate the effect of BDNF gene alleles on the risk of CKD in dominant, recessive, and additive models. A two-sided p value < 0.05 was considered to indicate statistical significance. STATA 16.1 (StataCorp, College Station, TX, USA) was used to conduct the statistical analyses.

## Results

### Part 1: analysis of the correlation between serum BDNF levels and CKD

Of the 480 enrolled participants, 87 (18.1%) were classified into the CKD group. The baseline characteristics of the participants grouped by their CKD status are shown in Table 1. The participants with CKD had a significantly lower eGFR than those without CKD. In addition, the participants with CKD were significantly older (69 ± 11 vs. 58 ± 11 years, P < 0.001) and had a significantly higher prevalence of hypertension (83% vs. 64%, P < 0.001), a higher systolic blood pressure (132 ± 18 vs. 126 ± 18 mmHg, P = 0.002), and a higher CRP (3.3 ± 3.0 vs. 2.2 ± 2.2 mg/L) than the participants without CKD. The participants with CKD had significantly lower serum BDNF at fasting than those without CKD (21.1 ± 7.4 vs. 25.2 ± 8.8 ng/mL, P < 0.001). Fasting glucose and insulin levels and HOMA-IR were not significantly different between the participants with and without CKD. The distributions of sex, current smoker, body mass index (BMI), diastolic blood pressure, UACR, HbA1c, lipid profile, and the use of antihypertensive and antiplatelet drugs were not significantly different between the participants with and without CKD.

**Table 1:** Baseline characteristics of the study participants by CKD status

	CKD (−) (n = 393)	CKD ( +) (n = 87)	P
Age (years)	58 (11)	69 (11)	< 0.001
Male (n, %)	323 (82.2)	70 (80.5)	0.700
Current smoker (n, %)	163 (41.5)	44 (50.6)	0.120
BMI (kg/m^2^)	26.2 (3.8)	25.4 (3.0)	0.068
Hypertension (n, %)	252 (64)	72 (83)	< 0.001
Systolic BP (mmHg)	126 (18)	132 (18)	0.002
Diastolic BP (mmHg)	74 (11)	73 (10)	0.100
Fasting BDNF (ng/mL)	25.2 (8.8)	21.1 (7.4)	< 0.001
Fasting glucose (mmol/L)	5.3 (0.7)	5.3 (1.0)	0.990
Fasting insulin (μIU/mL)	12.1 (14.1)	11.2 (7.7)	0.560
HOMA-IR	3.0 (4.3)	2.7 (2.1)	0.600
OGTT at 30 min			
BDNF (ng/mL)	19.9 (9.2)	18.2 (8.6)	0.120
Glucose (mmol/L)	9.3 (1.6)	9.1 (1.6)	0.430
Insulin (μIU/mL)	79.0 (80.9)	71.8 (49.9)	0.430
OGTT at 120 min			
BDNF (ng/mL)	17.9 (7.6)	15.4 (6.0)	0.004
Glucose (mmol/L)	8.1 (2.6)	8.7 (2.7)	0.052
Insulin (μIU/mL)	81.9 (80.8)	88.4 (68.6)	0.480
AUC during OGTT			
BDNF (ng/mL) × hr	39.6 (13.8)	35.0 (11.6)	0.004
Glucose (mmol/L) × hr	16.7 (3.1)	17.0 (3.1)	0.400
Insulin (μIU/mL) × hr	143.5 (127.8)	141.0 (83.9)	0.860
HbA1c (%)	5.8 (0.6)	5.9 (0.6)	0.580
eGFR (mL/min/1.73 m^2^)	84.1 (15.6)	47.3 (11.9)	< 0.001
Urine albumin-creatinine ratio (mg/g)	28.0 (97.7)	57.4 (146.6)	0.076
C-reactive protein (mg/L)	2.2 (2.2)	3.3 (3.0)	< 0.001
Lipid profile			
Total cholesterol (mmol/L)	4.4 (1.0)	4.3 (0.9)	0.590
HDL cholesterol (mmol/L)	1.2 (0.3)	1.2 (0.3)	0.480
Triglycerides (mmol/L)	1.5 (0.8)	1.5 (0.9)	0.740
Antiplatelet drugs (n, %)	374 (95.2)	82 (94.3)	0.720
Antihypertensive drugs (n, %)	338 (86.0)	78 (89.7)	0.360
ACEI or ARB	220 (56.0)	49 (56.3)	0.950
α-blocker	17 (4.3)	6 (6.9)	0.310
β-blocker	116 (29.5)	28 (32.2)	0.620
CCB	198 (50.4)	50 (57.5)	0.230
Diuretics	59 (15.0)	20 (23.0)	0.069

Continuous data are expressed as the mean (standard deviation). Categorical data are expressed as the number (percentage)

*ACEI* angiotensin-converting enzyme inhibitor, *ARB* angiotensin receptor blocker, *AUC* area under the curve, *BDNF* brain-derived neurotrophic factor, *BMI* body mass index, *CCB* calcium channel blocker, *CKD* chronic kidney disease, *eGFR* estimated glomerular filtration rate, *HbA1c* hemoglobin A1c, *HDL* high-density lipoprotein, *HOMA-IR* homeostatic model assessment of insulin resistance, *OGTT* oral glucose tolerance test

Measurements taken 30 min after glucose intake show that serum BDNF was not significantly different between the participants with and without CKD (P = 0.120). Likewise, at this time point glucose and insulin levels did not differ significantly between these two groups. Measurements taken 120 min after glucose intake show that the participants with CKD had significantly lower serum BDNF levels than those without CKD (15.4 ± 6.0 vs. 17.9 ± 7.6 ng/mL, P = 0.004), whereas the corresponding glucose and insulin levels did not differ significantly between these two groups. We calculated the AUC of BDNF, glucose, and insulin during the 120-min OGTT. The AUC of BDNF was significantly lower in participants with CKD than in those without CKD (35.0 ± 11.6 vs. 39.6 ± 13.8 mmol/L × hr, P = 0.004). However, the AUC values of glucose and insulin were still not significantly different between the participants with and without CKD. Table 2 shows that eGFR was significantly correlated with fasting BDNF and the AUC of BDNF but not with BDNF at 30 min or 120 min.

**Table 2:** Correlation between estimated glomerular filtration rate (eGFR) and brain-derived neurotrophic factor (BDNF) profile

	eGFR	
	Correlation coefficient	P
Fasting BDNF	0.194	< 0.001
BDNF at 30 min	0.062	0.172
BDNF at 120 min	0.076	0.097
AUC of BDNF	0.104	0.023

AUC = area under the curve

**Fig. 1:**
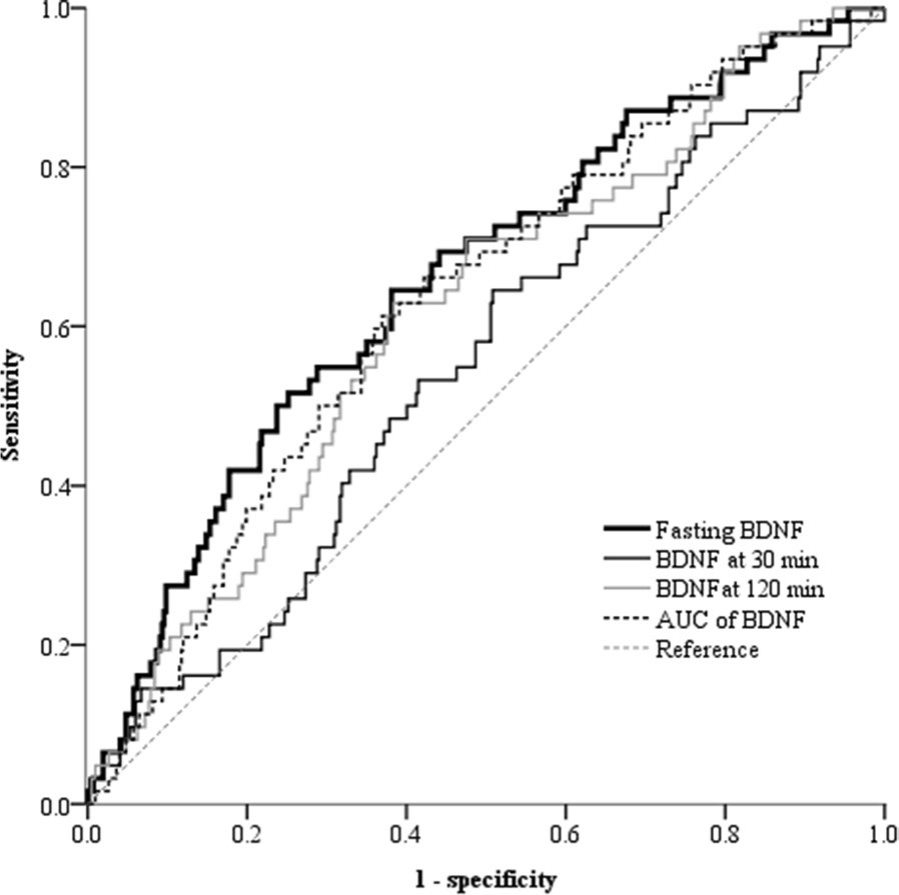
Receiver operating characteristic (ROC) curves for differentiating CKD based on serum brain-derived neurotrophic factor (BDNF) levels at fasting (fasting BDNF), 30 min (BDNF at 30 min), and 120 min (BDNF at 120 min), and for calculating the area under the curve (AUC) of BDNF during the oral glucose tolerance test (OGTT)

As shown in Fig. 1, the area under the ROC curve for differentiating CKD was largest using fasting BDNF (0.645, 95% CI: 0.583‒0.707) compared to BDNF at 30 min (0.547, 95% CI: 0.481‒0.612), BDNF at 120 min (0.598, 95% CI: 0.536‒0.661), and the AUC of BDNF (0.599, 95% CI: 0.537‒0.660). As shown in Table 3, participants in the higher quartile of fasting BDNF had the lower prevalence of CKD (P value for trend  < 0.001). Moreover, the participants in the lowest quartile of fasting BDNF were more likely to be older (P value for trend  < 0.001) and male (P value for trend = 0.023) and to have hypertension (P value for trend = 0.007), reduced BMI (P value for trend = 0.014), reduced diastolic blood pressure (P value for trend = 0.001), increased UACR (P value for trend = 0.009), increased CRP (P value for trend  < 0.001), decreased total cholesterol (P value for trend = 0.005), and decreased triglycerides (P value for trend  < 0.001).

**Table 3:** Baseline characteristics of the study participants by quartiles of fasting BDNF level

	Quartile 1 n = 120 (≤ 18.80 ng/mL)	Quartile 2 n = 120 (18.81‒23.69 ng/mL)	Quartile 3 n = 120 (23.70‒28.72 ng/mL)	Quartile 4 n = 120 (≥ 28.73 ng/mL)	*P*
CKD (n, %)	34 (28.3)	24 (20.0)	19 (15.8)	10 (8.3)	< 0.001
Age (years)	64 (13)	60 (11)	61 (11)	57 (12)	< 0.001
Male (n, %)	105 (87.5)	98 (81.7)	100 (83.3)	90 (75.0)	0.023
Current smoker (n, %)	51 (42.5)	54 (45.0)	47 (39.2)	55 (45.8)	0.837
BMI (kg/m^2^)	25.4 (3.3)	25.7 (3.0)	26.4 (4.1)	26.8 (4.3)	0.014
Hypertension (n, %)	92 (76.7)	82 (68.3)	77 (64.2)	73 (60.8)	0.007
Systolic BP (mmHg)	127 (18)	126 (17)	126 (21)	129 (16)	0.609
Diastolic BP (mmHg)	72 (11)	74 (11)	75 (11)	77 (9)	0.001
HbA1c (%)	5.9 (0.6)	5.8 (0.5)	5.9 (0.6)	5.8 (0.6)	0.531
Fasting glucose (mmol/L)	5.4 (0.9)	5.2 (0.5)	5.3 (0.8)	5.4 (0.7)	0.829
HOMA-IR	2.8 (3.5)	2.5 (2.2)	2.8 (2.6)	3.5 (6.3)	0.120
Urine albumin-creatinine ratio (mg/g)	39.1 (106.5)	22.2 (56.8)	33.5 (124.0)	38.4 (131.8)	0.009
C-reactive protein (mg/L)	3.1 (2.6)	2.4 (2.5)	2.1 (2.2)	2.03 (2.3)	< 0.001
Lipid profile					
Total cholesterol (mmol/L)	4.2 (0.8)	4.4 (1.0)	4.4 (1.1)	4.6 (1.0)	0.005
HDL cholesterol (mmol/L)	1.2 (0.3)	1.2 (0.3)	1.2 (0.3)	1.2 (0.3)	0.328
Triglycerides (mmol/L)	1.3 (0.6)	1.5 (0.8)	1.5 (0.8)	1.7 (1.0)	< 0.001

Continuous data are expressed as the mean (standard deviation). Categorical data are expressed as number (percentage)

*CKD* chronic kidney disease, *BMI* body mass index, *BDNF* brain-derived neurotrophic factor, *HbA1c* hemoglobin A1c. *HOMA-IR* homeostatic model assessment of insulin resistance, *HDL* high-density lipoprotein

Table 4 shows the OR of CKD in the three higher quartiles of fasting BDNF compared to the lowest quartile (reference group). The participants in the highest fasting BDNF quartile had a significantly lower CKD prevalence than those in the lowest quartile, with an OR of 0.30 (95% CI 0.12‒0.71) after adjusting for potential confounding variables (Additional file 1: Fig. S1a).

**Table 4:** Odds ratios (95% CI) for chronic kidney disease (CKD) by quartiles of fasting brain-derived neurotrophic factor levels

	Quartile 1 n = 120 (≤ 18.80 ng/mL)	Quartile 2 n = 120 (18.81‒23.68 ng/mL)	Quartile 3 n = 120 (23.70‒28.69 ng/mL)	Quartile 4 n = 120 (≥ 28.77 ng/mL)
CKD/non-CKD	34/86	24/96	19/101	10/110
Model 1	1.00 (reference)	0.63 (0.35–1.15)	0.48 (0.25–0.89)	0.23 (0.11–0.49)
Model 2	1.00 (reference)	0.85 (0.44–1.62)	0.58 (0.29–1.14)	0.33 (0.15–0.75)
Model 3	1.00 (reference)	0.80 (0.40–1.57)	0.61 (0.30–1.24)	0.30 (0.12–0.71)

Model 1: Crude

Model 2: Adjusted for age and sex

Model 3: Adjusted for age, sex, body mass index, coronary artery disease, hypertension, current smoking, hemoglobin A1c, homeostatic model assessment of insulin resistance, urine albumin-creatinine ratio, C-reactive protein, total cholesterol, and triglycerides

The prevalence of CKD was not significantly associated with quartiles of BDNF levels at 30 min (P value for trend = 0.231, Additional file 2: Table S1). The OR of CKD in the highest quartile of BDNF levels at 30 min (1.03, 95% CI 0.47‒2.24) was not significantly different from that in the lowest quartile after adjusting for associated risk factors (Additional file 3: Table 2, Additional file 1: Fig. S1b). Although the prevalence of CKD was significantly associated with the quartiles of BDNF levels at 120 min (P value for trend = 0.004, Additional file 4: Table S3), the OR of CKD in the highest quartile of BDNF levels at 120 min (0.47, 95% CI 0.21‒1.06) was not significantly different from that in the lowest quartile after adjustment for associated risk factors (Additional file 5: Table S4, Additional file 1: Fig. S1c).

### Part 2: genetic analysis of the association between BDNF SNPs and CKD

There were 167 participants aged < 60 years with CKD in the case group and 1105 participants aged  ≥ 60 years without CKD in the control group. Except for rs77351929, the genotype frequencies of the other four assessed SNPs were in Hardy–Weinberg equilibrium in the case–control analyses. The SNPs including rs12098908, rs12577517, and rs72891405 were significantly associated with CKD (P < 0.01). Among all 3003 enrolled participants, all the risk SNPs detected in the case–control analysis were significantly associated with CKD in the dominant model, including rs12098908 with OR = 0.733 (95% CI 0.601‒0.895), rs12577517 with OR = 0.802 (95% CI 0.663‒0.969), and rs72891405 with OR = 0.800 (95% CI 0.662‒0.967), after adjusting for age and sex (Table 5).

**Table 5:** Genotype frequencies and effects of BDNF-associated variants on CKD

	Case (n = 167) and control (n = 1105)*					All enrolled participants (N = 2997)^#^		
SNP	MAF	Genotype	Case N (%)	Control N (%)	P	Model^†^	OR/B^‡^ 95% CI	P
rs12098908	0.142	CC	135 (83.3%)	778 (72.4%)	0.004	Dominant	0.733 (0.601, 0.895)	0.002
		CT	22 (13.6%)	272 (25.3%)		Recessive	0.710 (0.386, 1.306)	0.271
		TT	5 (3.1%)	24 (2.2%)		Additive	− 0.047 (− 0.079, − 0.016)	0.003
rs12577517	0.151	AA	135 (80.8%)	783 (70.9%)	0.006	Dominant	0.802 (0.663, 0.969)	0.022
		AG	26 (15.6%)	297 (26.9%)		Recessive	0.847 (0.479, 1.500)	0.570
		GG	6 (3.6%)	25 (2.3%)		Additive	− 0.032 (− 0.063, − 0.002)	0.039
rs6265	0.500	CC	45 (27.1%)	277 (25.0%)	0.309			
		CT	73 (44.0%)	555 (50.2%)				
		TT	48 (28.9%)	274 (24.8%)				
rs72891405	0.151	CC	135 (80.8%)	783 (70.9%)	0.006	Dominant	0.800 (0.662, 0.967)	0.021
		CT	26 (15.6%)	297 (26.9%)		Recessive	0.846 (0.478, 1.498)	0.567
		TT	6 (3.6%)	25 (2.3%)		Additive	− 0.033 (− 0.063, − 0.002)	0.037

^*^Case means age < 60 years with CKD; control means age ≥ 60 years but without CKD

^#^Analyses were not performed on rs6265 as there was no significant difference in case–control assessment

^†^Adjusted for age and sex. ^‡^OR = odds ratio for the dominant and recessive models, and B = regression coefficient in the additive model

*BDNF* brain-derived neurotrophic factor, *CKD* chronic kidney disease, *MAF* minor allele frequency, *SNP* single nucleotide polymorphism

## Discussion

The main finding of our study was that a higher fasting serum BDNF level was associated with a significantly lower prevalence of CKD. The inverse association between fasting BDNF and CKD implies that BDNF might have a renoprotective effect in participants without known DM. Consistent with our results, Kurajoh et al. [18] reported that lower circulating BDNF was an independent predictor of incident CKD among patients at risk of cardiovascular disease in a longitudinal study.

Considering the known association between BDNF and energy homeostasis [30], the strength of our study is the comparison of the effect of serum BDNF after oral glucose intake on CKD with that of fasting serum BDNF. Our previous studies found that the AUC of serum BDNF levels during the OGTT was strongly associated with a reduction in central pulse pressure [31], and that it better predicted the development of cardiovascular events than fasting BDNF [22]. However, the results of the present study revealed that dynamic serum BDNF levels after oral glucose intake did not provide more information on CKD than fasting BDNF levels.

The mechanisms underlying the inverse association between BDNF levels and CKD prevalence have been investigated in several in vitro and animal studies. BDNF was reported to increase both the length and number of podocyte cell processes in an in vitro model, and exogenous BDNF administration was found to improve glomerular lesions and repair podocyte damage in an in vivo mouse model of adriamycin-induced nephropathy [17]. Additionally, endogenous BDNF has been reported to protect the kidney from endoplasmic reticulum stress-induced apoptosis in a mouse study [32].

The expression of both BDNF and the TrkB receptor has been detected in the kidney [17, 33–35]. BDNF probably affects renal physiology through TrkB signaling, which has been suggested to play a key role in early renal mesenchymal cell differentiation [36]. Indeed, the importance of TrkB in kidney function and structure is supported by evidence that TrkB deficiency in mice is associated with overabundance of extraglomerular mesangial cells, decreased glomerular area, and absence of the macula densa [33].

Several lines of evidence indicate that circulating BDNF levels are inversely associated with adverse cardiovascular outcomes. A lower serum BDNF level predicted a greater cardiovascular risk [21], while several genotypic variants of BDNF were distinctly associated with ischemic stroke [24]. Although these results overall support a cardioprotective effect of BDNF [37], the authors did not characterize either renal function or CKD status in the study participants. Notably, serum BDNF level before percutaneous coronary intervention (PCI) was found to correlate with the post-PCI reduction in renalase, predictive in turn of long-term cardiovascular events [38]. Therefore, BDNF might prevent cardiorenal syndrome [39, 40].

Interestingly, since TrkB inhibition was shown to induce podocyte dedifferentiation and a strong correlation was detected between mRNA levels of BDNF in the urine cells and various indicators of kidney injury in CKD patients, it was suggested that BDNF in urine cells could serve as a biomarker of CKD [41]. Compared with an age-matched control group, patients with end-stage renal disease had a significantly lower serum BDNF level due to higher inflammation and oxidative stress after dialysis [42]. Considering that both the incidence and prevalence of CKD are high in Taiwan and that CKD has become a serious public health burden [43, 44], the association between BDNF and CKD might be valuable evidence for developing future clinical and public health interventions.

Several studies reported the association between the rs6265 polymorphism, an exonic variant in the BDNF gene, and both neuropsychiatric disorders and cardiovascular disease [26, 45, 46]. However, investigations into the association between rs6265 and CKD are lacking. In the present study, the risk allele of rs6265 was not significantly prevalent in participants with susceptibility to CKD, defined as eGFR < 60 mL/min/1.73m^2^ before 60 years of age compared to those with eGFR ≥ 60 mL/min/1.73 m^2^ even after 60 years of age. On the other hand, several intronic BDNF variants, namely rs12098908, rs12577517, and rs72891405, were significantly more prevalent in cases who were susceptible to CKD than in controls without CKD. Among all participants included in the genetic assessments, these intronic BDNF variants provided a protective benefit with an OR of approximately 0.7–0.8 against CKD after adjusting for age and sex. In line with our findings, the intronic variants of BDNF were reported to be associated with disease severity presenting as proteinuria in patients with IgA nephropathy [47]. Of note, it has been reported that minor alleles of BDNF variants are associated with increased serum BDNF levels [21]. In this regard, a caveat of our study is that we did not compare serum BDNF levels between participants with different genotypes. Hence, further investigations on the link between BDNF polymorphisms and CKD are warranted.

There are several limitations in the present study. First, the clinical characteristics were collected only at the baseline measurement, and the results cannot ultimately determine causality because of the cross-sectional study design. Second, we did not directly investigate the molecular mechanisms linking BDNF to renal pathophysiology. Third, we did not assess the participants’ appetite in the present study. Stanek et al. [48] reported that serum BDNF might be inversely correlated with appetite. Further studies on the relationship between anorexigenic manifestations and postprandial BDNF levels in patients with CKD are thus needed. Fourth, this study was conducted in a single-hospital population, hence the results might not be widely generalizable. Finally, to make our analysis more efficient and minimize confounding, we excluded participants who had known DM. This decision was based on both DM being an important risk factor for CKD [49], and evidence of decreased circulating BDNF in patients with DM [50]. Therefore, our findings might not be applicable to the population with diabetes.

## Conclusions

A higher fasting serum BDNF level is significantly associated with a lower prevalence of CKD in patients without known DM. BDNF gene SNPs were found to be associated with CKD. Future larger studies are warranted to further explore the underlying mechanism and the and the causal relationship between BDNF and CKD.

## Supplementary Information

**Additional file 1: Figure S1.** Odds ratios (95% CI) for chronic kidney disease by quartiles of brain-derived neurotrophic factor (BDNF) levels at fasting (a), 30 min (b), and 120 min (c) during the OGTT after adjustment for age, sex, body mass index, coronary artery disease, hypertension, current smoking, smoking, hemoglobin A1c, homeostatic model assessment of insulin resistance, urine albumin-creatinine ratio, C-reactive protein, total cholesterol and, triglycerides. (OGTT = oral glucose tolerance test).

**Additional file 2: Table S1.** Baseline characteristics of the study participants by quartiles of BDNF levels at 30 min.

**Additional file 3: Table S2.** Odds ratios (95% CI) for chronic kidney disease (CKD) by quartiles of brain-derived neurotrophic factor levels at 30 min.

**Additional file 4: Table S3.** Baseline characteristics of the study participants by quartiles of BDNF levels at 120 min.

**Additional file 5: Table S4.** Odds ratios (95% CI) for chronic kidney disease (CKD) by quartiles of brain derived neurotrophic factor levels at 120 min.

## Data Availability

The datasets used and/or analyzed during the current study are available from the corresponding author on reasonable request.

## Abbreviations

AUC: Area under the curve
BDNF: Brain-derived neurotrophic factor
BMI: Body mass index
CI: Confidence interval
CKD: Chronic kidney disease
CRP: C-reactive protein
CV: Coefficient of variation
DM: Diabetes mellitus
eGFR: Estimated glomerular filtration rate
HbA1c: Hemoglobin A1c
HOMA-IR: Homeostasis model assessment of insulin resistance
OGTT: Oral glucose tolerance test
OR: Odds ratio
PCI: Percutaneous coronary intervention
ROC: Receiver operating characteristic
SNPs: Single-nucleotide polymorphisms
TrkB: Tyrosine kinase receptor B
UACR: Urine albumin-creatinine ratio
