# Supplementary material for: Brain-derived neurotrophic factor associated with kidney function
Source: Diabetol Metab Syndr. 2023 Feb 13;15:16. doi: 10.1186/s13098-023-00991-5 (PMC9926783; doi:10.1186/s13098-023-00991-5)

**Additional file Figure 1.** Odds ratios (95% CI) for chronic kidney disease by quartiles of brain-derived neurotrophic factor (BDNF) levels at fasting (a), 30 min (b), and 120 min (c) during the OGTT after adjustment for age, sex, body mass index, coronary artery disease, hypertension, current smoking, smoking, hemoglobin A1c, homeostatic model assessment of insulin resistance, urine albumin-creatinine ratio, C-reactive protein, total cholesterol and, triglycerides. (OGTT = oral glucose tolerance test)

(a)


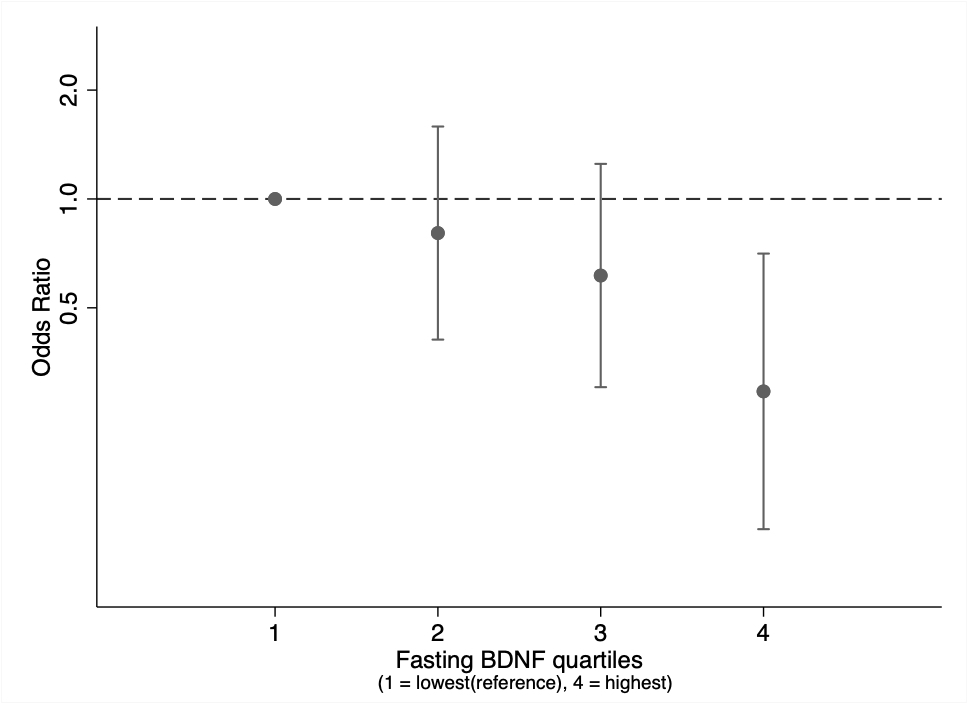


(b)


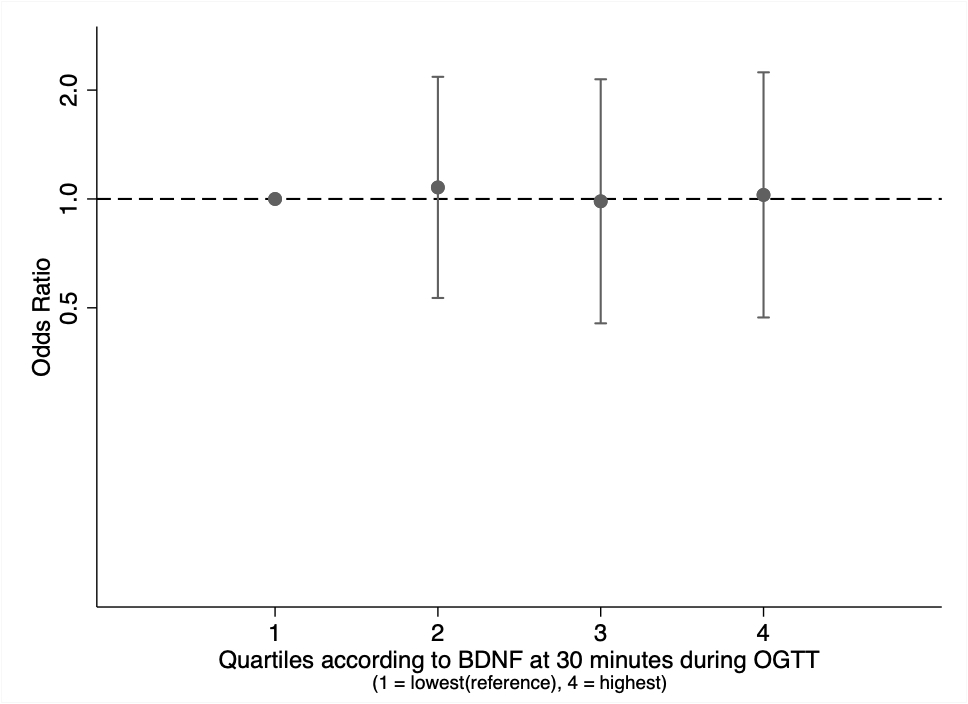


(c)


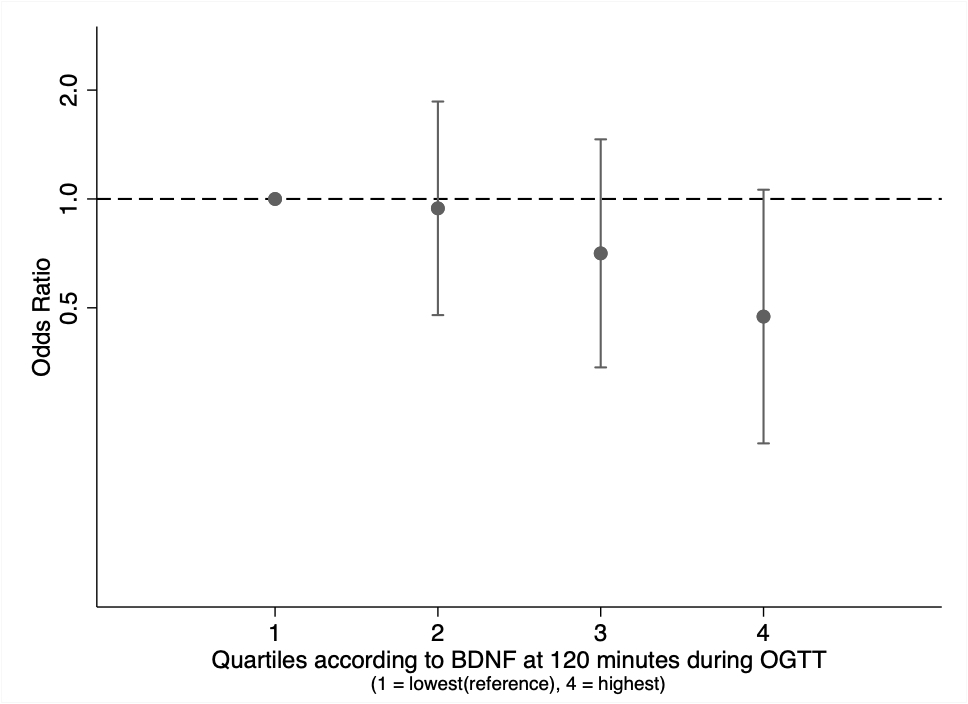

Supplement: Supplementary file 1 — Additional file 1: Figure S1. Odds ratios (95% CI) for chronic kidney disease by quartiles of brain-derived neurotrophic factor (BDNF) levels at fasting (a), 30 min (b), and 120 min (c) during the OGTT after adjustment for age, sex, body mass index, coronary artery disease, hypertension, current smoking, smoking, hemoglobin A1c, homeostatic model assessment of insulin resistance, urine albumin-creatinine ratio, C-reactive protein, total cholesterol and, triglycerides. (OGTT = oral glucose tolerance test). [file 13098_2023_991_MOESM1_ESM.docx]
