# Supplementary material for: Brain-derived neurotrophic factor associated with kidney function
Source: Diabetol Metab Syndr. 2023 Feb 13;15:16. doi: 10.1186/s13098-023-00991-5 (PMC9926783; doi:10.1186/s13098-023-00991-5)
Supplement: Supplementary file 2 — Additional file 2: Table S1. Baseline characteristics of the study participants by quartiles of BDNF levels at 30 min. [file 13098_2023_991_MOESM2_ESM.docx]

| Additional file Table 1. Baseline characteristics of the study participants by quartiles of BDNF levels at 30 min. | | | | | | | | | |
| --- | --- | --- | --- | --- | --- | --- | --- | --- | --- |
|  | Quartile 1  n = 121  (≤12.97 ng/mL) | | Quartile 2  n= 119  (12.98‒18.64 ng/mL) | | Quartile 3  n = 120  (18.65‒25.26 ng/mL) | | Quartile 4  n = 120  (≥25.27 ng/mL) | | P |
| CKD (n, %) | 24 | (19.8) | 27 | (22.7) | 16 | (13.3) | 20 | (16.7) | 0.231 |
| Age (years) | 61.5 | (11.6) | 62.8 | (12.4) | 58.1 | (10.3) | 59.2 | (12.8) | 0.013 |
| Male (n, %) | 97 | (80.2) | 98 | (82.4) | 102 | (85.0) | 96 | (80.0) | 0.889 |
| Current smoker (n, %) | 60 | (49.6) | 58 | (48.7) | 43 | (35.8) | 46 | (38.3) | 0.021 |
| BMI (kg/m^2^) | 25.5 | (3.5) | 25.9 | (3.6) | 25.9 | (3.2) | 27.0 | (4.4) | 0.011 |
| Hypertension (n, %) | 90 | (74.4) | 90 | (75.6) | 73 | (60.8) | 71 | (59.2) | 0.002 |
| Systolic BP (mmHg) | 126.3 | (19.7) | 127.7 | (20.9) | 126.2 | (15.4) | 128.2 | (15.7) | 0.273 |
| Diastolic BP (mmHg) | 72.9 | (9.9) | 74.4 | (12.2) | 74.2 | (9.6) | 75.5 | (10.4) | 0.042 |
| HbA1c (%) | 5.7 | (0.6) | 5.8 | (0.5) | 5.9 | (0.5) | 5.9 | (0.7) | 0.002 |
| Fasting glucose (mmol/L) | 5.2 | (0.7) | 5.4 | (0.9) | 5.3 | (0.6) | 5.4 | (0.8) | 0.336 |
| HOMA-IR | 2.5 | (1.8) | 2.5 | (1.8) | 3.0 | (3.5) | 3.7 | (6.6) | 0.269 |
| Urine albumin-creatinine ratio (mg/g) | 32.5 | (68.2) | 34.6 | (108.3) | 21.2 | (80.4) | 44.9 | (156.2) | 0.028 |
| C-reactive protein (mg/L) | 3.0 | (2.5) | 2.5 | (2.5) | 2.2 | (2.3) | 1.9 | (2.4) | <0.001 |
| Lipid profile |  |  |  |  |  |  |  |  |  |
| Total cholesterol (mmol/L) | 4.2 | (1.0) | 4.4 | (1.0) | 4.4 | (1.0) | 4.6 | (1.0) | <0.001 |
| HDL cholesterol (mmol/L) | 1.2 | (0.3) | 1.3 | (0.3) | 1.2 | (0.2) | 1.2 | (0.3) | 0.936 |
| Triglycerides (mmol/L) | 1.3 | (0.7) | 1.5 | (0.8) | 1.5 | (0.7) | 1.7 | (1.1) | <0.001 |
| Continuous data are expressed as the mean (standard deviation). Categorical data are expressed as number (percentage).  CKD = chronic kidney disease, BDNF = brain-derived neurotrophic factor, BMI = body mass index, HbA1c = hemoglobin A1c, HDL = high-density lipoprotein, and HOMA-IR = homeostatic model assessment of insulin resistance. | | | | | | | | | |
