# Supplementary material for: Brain-derived neurotrophic factor associated with kidney function
Source: Diabetol Metab Syndr. 2023 Feb 13;15:16. doi: 10.1186/s13098-023-00991-5 (PMC9926783; doi:10.1186/s13098-023-00991-5)
Supplement: Supplementary file 3 — Additional file 3: Table S2. Odds ratios (95% CI) for chronic kidney disease (CKD) by quartiles of brain-derived neurotrophic factor levels at 30 min. [file 13098_2023_991_MOESM3_ESM.docx]

| Additional fileTable 2. Odds ratios (95% CI) for chronic kidney disease (CKD) by quartiles of brain-derived neurotrophic factor levels at 30 min | | | | |
| --- | --- | --- | --- | --- |
|  | Quartile 1  n = 121  (≤12.97 ng/mL) | Quartile 2  n= 119  (12.98‒18.64 ng/mL) | Quartile 3  n = 120  (18.65‒25.26 ng/mL) | Quartile 4  n = 120  (≥25.27 ng/mL) |
| CKD/nonCKD | 24/97 | 27/92 | 16/104 | 20/100 |
| Model 1 | 1.00 (reference) | 1.19 (0.64-2.20) | 0.62 (0.31-1.24) | 0.81 (0.42-1.56) |
| Model 2 | 1.00 (reference) | 1.06 (0.54-2.06) | 0.82 (0.39-1.70) | 0.89 (0.44-1.90) |
| Model 3 | 1.00 (reference) | 1.08 (0.53-2.18) | 0.99 (0.45-2.14) | 1.03 (0.47-2.24) |
| Model 1: Crude.  Model 2: Adjusted for age and sex.  Model 3: Adjusted for age, sex, body mass index, coronary artery disease, hypertension, current smoking, hemoglobin A1c, homeostatic model assessment of insulin resistance, urine albumin-creatinine ratio, C-reactive protein, total cholesterol, and triglycerides. | | | | |
