# Supplementary material for: Brain-derived neurotrophic factor associated with kidney function
Source: Diabetol Metab Syndr. 2023 Feb 13;15:16. doi: 10.1186/s13098-023-00991-5 (PMC9926783; doi:10.1186/s13098-023-00991-5)
Supplement: Supplementary file 4 — Additional file 4: Table S3. Baseline characteristics of the study participants by quartiles of BDNF levels at 120 min. [file 13098_2023_991_MOESM4_ESM.docx]

| Additional fileTable 3. Baseline characteristics of the study participants by quartiles of BDNF levels at 120 min. | | | | | | | | | |
| --- | --- | --- | --- | --- | --- | --- | --- | --- | --- |
|  | Quartile 1  n = 120  (≤12.51 ng/mL) | | Quartile 2  n= 120  (12.52‒16.82 ng/mL) | | Quartile 3  n = 120  (16.83‒21.74 ng/mL) | | Quartile 4  n = 120  (≥21.75 ng/mL) | | P |
| CKD (n, %) | 29 | (24.2) | 26 | (21.7) | 19 | (15.8) | 13 | (10.8) | 0.004 |
| Age (years) | 62.5 | (11.9) | 61.6 | (12.1) | 59.8 | (11.4) | 57.8 | (11.9) | <0.001 |
| Male (n, %) | 97 | (80.8) | 97 | (80.8) | 99 | (82.5) | 100 | (83.3) | 0.560 |
| Current smoker (n, %) | 60 | (50.0) | 54 | (45.0) | 50 | (41.7) | 43 | (35.8) | 0.023 |
| BMI (kg/m^2^) | 25.6 | (3.4) | 25.4 | (3.4) | 26.4 | (3.4) | 26.9 | (4.4) | 0.012 |
| Hypertension (n, %) | 90 | (75.0) | 82 | (68.3) | 75 | (62.5) | 77 | (64.2) | 0.045 |
| Systolic BP (mmHg) | 127.5 | (19.2) | 124.2 | (18.6) | 128.3 | (15.8) | 128.4 | (18.5) | 0.543 |
| Diastolic BP (mmHg) | 73.2 | (10.2) | 72.9 | (10.2) | 75.0 | (10.4) | 75.9 | (11.4) | 0.026 |
| HbA1c (%) | 5.8 | (0.6) | 5.8 | (0.5) | 5.9 | (0.6) | 5.8 | (0.5) | 0.168 |
| Fasting glucose (mmol/L) | 5.4 | (0.9) | 5.3 | (0.6) | 5.3 | (0.7) | 5.3 | (0.6) | 0.814 |
| HOMA-IR | 2.4 | (1.7) | 2.6 | (2.1) | 3.0 | (3.6) | 3.7 | (6.5) | 0.047 |
| Urine albumin-creatinine ratio (mg/g) | 36.1 | (106.6) | 38.9 | (125.8) | 34.0 | (115.3) | 24.2 | (82.9) | 0.197 |
| C-reactive protein (mg/L) | 2.9 | (2.5) | 2.1 | (2.6) | 2.4 | (2.6) | 2.1 | (2.0) | 0.254 |
| Lipid profiles |  |  |  |  |  |  |  |  |  |
| Total cholesterol (mmol/L) | 4.3 | (0.9) | 4.4 | (1.0) | 4.5 | (1.0) | 4.5 | (0.9) | 0.020 |
| HDL cholesterol (mmol/L) | 1.2 | (0.3) | 1.3 | (0.3) | 1.2 | (0.3) | 1.2 | (0.3) | 0.166 |
| Triglycerides (mmol/L) | 1.3 | (0.6) | 1.4 | (0.7) | 1.6 | (0.9) | 1.7 | (1.1) | <0.001 |
| Continuous data are expressed as the mean (standard deviation). Categorical data are expressed as number (percentage).  CKD = chronic kidney disease, BDNF = brain-derived neurotrophic factor, BMI = body mass index, HbA1c = hemoglobin A1c, HDL = high-density lipoprotein, and HOMA-IR = homeostatic model assessment of insulin resistance. | | | | | | | | | |
