# Supplementary material for: Brain-derived neurotrophic factor associated with kidney function
Source: Diabetol Metab Syndr. 2023 Feb 13;15:16. doi: 10.1186/s13098-023-00991-5 (PMC9926783; doi:10.1186/s13098-023-00991-5)
Supplement: Supplementary file 5 — Additional file 5: Table S4. Odds ratios (95% CI) for chronic kidney disease (CKD) by quartiles of brain derived neurotrophic factor levels at 120 min. [file 13098_2023_991_MOESM5_ESM.docx]

| Additional fileTable 4. Odds ratios (95% CI) for chronic kidney disease (CKD) by quartiles of brain derived neurotrophic factor levels at 120 min | | | | |
| --- | --- | --- | --- | --- |
|  | Quartile 1  n = 120  (<12.51 ng/mL) | Quartile 2  n= 120  (12.52-16.82 ng/mL) | Quartile 3  n = 120  (16.83-21.74 ng/mL) | Quartile 4  n = 120  (>21.75 ng/mL) |
| CKD/non-CKD | 29/91 | 26/94 | 19/101 | 13/107 |
| Model 1 | 1.00 (reference) | 0.87 (0.48-1.59) | 0.59 (0.31-1.12) | 0.38 (0.19-0.78) |
| Model 2 | 1.00 (reference) | 0.90 (0.47-1.73) | 0.69 (0.35-1.38) | 0.49 (0.23-1.03) |
| Model 3 | 1.00 (reference) | 0.94 (0.48-1.86) | 0.71 (0.34-1.46) | 0.47 (0.21-1.06) |
| Model 1: Crude.  Model 2: adjusted for age and sex.  Model 3: adjusted for age, sex, body mass index, coronary artery disease, hypertension, current smoking, hemoglobin A1c, homeostatic model assessment of insulin resistance, urine albumin-creatinine ratio, C-reactive protein, total cholesterol and, triglycerides. | | | | |
